# Ewing Sarcoma of the Kidney, a Rare Entity: Case Report

**DOI:** 10.1155/carm/2598222

**Published:** 2025-07-26

**Authors:** Alina Baral, C. B. Pun, Binita Goyal, Subha Lamichhane

**Affiliations:** ^1^Department of Pathology, Bharatpur Hospital, Chitwan, Bharatpur, Nepal; ^2^Department of Pathology, College of Medical Sciences, Chitwan, Bharatpur, Nepal

**Keywords:** Ewing sarcoma, histopathology, kidney

## Abstract

Ewing sarcoma is a small round cell tumor of uncertain differentiation, primarily originating in bone in children and adolescents. Ewing sarcoma of the kidney is a rare occurrence and follows an aggressive course with early metastasis. Herein, we present a case of a 16-year-old male presenting with abdominal pain and lump. He underwent nephrectomy and histopathological diagnosis of small round cell tumor with differential diagnosis of Ewing sarcoma was made which was further confirmed by immunohistochemistry. Thus, Ewing sarcoma must also be taken into consideration while dealing with tumors of the kidney in young age group.

## 1. Introduction

Ewing sarcoma (ES) is a small round cell tumor or sarcoma with FET family gene fusion and ETS family transcription. It is the second most common malignant bone tumor in children and adolescent [1]. Extraskeletal involvement might be seen in soft tissue and viscera, but is very rare, and renal (kidney) ES is one of the rare entities with limited reported cases [2]. It is hypothesized that renal Ewing might originate from neural cells that invaginate into the kidney during development, or embryonic neural crest cells do migrate into the kidney and undergo tumorigenesis [3, 4]. Primary ES of the kidney was first described in 1975 by Seemayer and colleagues [3].

## 2. Case Discussion

A 16-year-old male presented with an abdominal mass and weight loss for three months at Urology OPD. Physical examination revealed a ballotable large mass on the left flank. CECT abdomen was done which showed heterogenous mass around 17 cm in left kidney with calcifications. Left radical nephrectomy was done and sent for histopathology.

**Figure 1:**
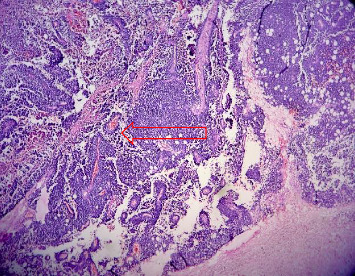
Small round blue tumor cells in sheets and perivascular pseudorosettes (marked with arrow), H&E stain, 10x magnification.

**Figure 2:**
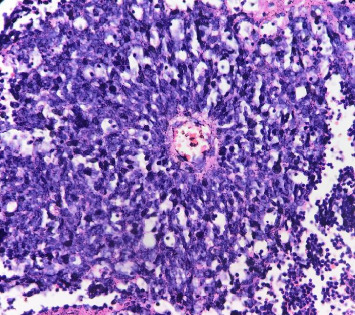
Hyperchromatic tumor cells with scant cytoplasm arranged around vessel, H&E stain, 40x magnification.

Gross examination revealed enlarged kidney with intact capsule. The cut surface showed a variegated, friable mass, gray-white in color, with areas of hemorrhage and necrosis involving the entire kidney, along with parenchymal destruction and effacement of cortex and medulla. On microscopy, the tumor revealed partially circumscribed lesion which was markedly cellular. It was composed of sheets of small round cells with indistinct cell borders and uniform round nuclei, showing finely dispersed chromatin, absent to inconspicuous nucleoli, and scant cytoplasm. Few vague papillary pattern and pseudorosettes are shown in Figures 1 and 2. Frequent mitotic figures, along with large areas of hemorrhage and necrosis, were discerned. Normal renal parenchyma was not identified histologically as well. Multiple sections were taken to rule out other epithelial or mesenchymal components. A histomorphological diagnosis of small round cell tumor with differential diagnosis of ES was rendered. It was further confirmed by immunohistochemistry with strong CD99 membranous positivity and FLI-1 positivity. After that, the patient was sent to a cancer center for chemotherapy. 2 cycles of chemotherapy (vincristine, doxorubicin, and cyclophosphamide [VAC]) were given but patient lost follow up after that. Thus outcome of patient could not be traced.

## 3. Discussion

ES and PNETs were formerly classified as two distinct pathologic entities. Owing to their similar histologic and cytogenetic characteristics, these tumors are now categorized under a spectrum of neoplastic diseases known as the ES family of tumors [5].

Primary ES of the kidney is an extremely rare entity and is typically seen in children and adolescents with a male predilection [6]. Similar was finding in our case. The clinical presentations include flank or abdominal pain, palpable mass, and hematuria [7, 8].

Primary ES of the kidney has propensity for early metastasis and follows an aggressive course [9].

No specific sign of ES of the kidney has been described on radiology. The imaging characteristics of most renal sarcomas are indistinguishable from those of renal cell carcinoma (RCC) and can radiologically masquerade as RCC [9].

Diagnosis of PNET is based on morphological, immunohistochemical, and genetic analyses. Morphologically small, round cells with hyperchromatic nuclei, scant to moderate cytoplasm, and occasional rosette-like structures are observed in PNET of the kidney [4]. Owing to age group, site, and histomorphology ES needs to be differentiated from other round cell tumors like rhabdomyosarcoma, Wilms tumor, neuroblastoma, lymphoma, desmoplastic small round cell tumor, etc. Multiple panel of immunohistochemistry markers (cytokeratin, WT1, synaptophysin, muscle-specific actin, desmin, EMA, myogenin, LCA, and CD45) is mandatory to exclude the diagnosis of other round cell tumors [10, 11].

Meanwhile, identification of the EWS/FLI-1 gene fusion by cytogenetics aids in confirming the diagnosis along with histopathology and immunohistochemistry [12].

ES are typically aggressive, involving the lymph nodes and metastasize to lung, bone, and liver. Median survival for advanced disease is only 5.6 months [8]. Multidimensional treatment approach of surgery and/or radiotherapy and chemotherapy is beneficial [8, 11]. The current regimens include alternating cycles of chemotherapy consisting of: VAC and ifosfamide-etoposide [13]. Extraskeletal Ewing's is a radiosensitive tumor but radiation therapy preferably plays role in the cases with unresectable disease [14].

## 4. Conclusion

ES of the kidney is a rare entity with presentation at young age and shows an aggressive disease course; thus, early diagnosis and multidimensional treatment are a must. Given its rarity, the presentation of the ES requires a diagnosis that relies on an integrated approach consisting of histomorphology, immunohistochemistry, and further confirmation by molecular-genetic testing whenever available.

## Data Availability

Data sharing is not applicable to this article as no datasets were generated or analysed during the current study.

## References

[1] Heare T., Hensley M. A., Dell’Orfano S. (2009). Bone Tumors: Osteosarcoma and Ewing’s Sarcoma. *Current Opinion in Pediatrics*.

[2] Celli R., Cai G. (2016). Ewing Sarcoma/Primitive Neuroectodermal Tumor of the Kidney: A Rare and Lethal Entity. *Archives of Pathology & Laboratory Medicine*.

[3] Seemayer T. A., Thelmo W. L., Bolande R. P., Wiglesworth F. W. (1975). Peripheral Neuroectodermal Tumors. *Perspectives in Pediatric Pathology*.

[4] Parham D. M., Roloson G. J., Feely M., Green D. M., Bridge J. A., Beckwith J. B. (2001). Primary Malignant Neuroepithelial Tumors of the Kidney: A Clinicopathologic Analysis of 146 Adult and Pediatric Cases From the National Wilms’ Tumor Study Group Pathology Center. *The American Journal of Surgical Pathology*.

[5] Lalwani N., Prasad S. R., Vikram R., Katabathina V., Shanbhogue A., Restrepo C. (2011). Pediatric and Adult Primary Sarcomas of the Kidney: A cross-sectional Imaging Review. *Acta Radiologica*.

[6] Kar S., Shah S., Menon M., Geetha N. (2021). Primary Ewing’s Sarcoma/Primitive Neuroectodermal Tumour of the Kidney: Report of Four Cases From a Tertiary Care Centre. *Current Problems in Cancer: Case Reports*.

[7] Choubey S., Pipara G., Kumar A. (2017). Ewings Sarcoma of the Kidney: A Rare Entity. *World Journal of Nephrology and Urology*.

[8] Hakky T. S., Gonzalvo A. A., Lockhart J. L., Rodriguez A. R. (2013). Primary Ewing Sarcoma of the Kidney: A Symptomatic Presentation and Review of the Literature. *Therapeutic Advances in Urology*.

[9] Mukkunda R., Venkitaraman R., Thway K. (2009). Primary Adult Renal Ewing’s Sarcoma: A Rare Entity. *Sarcoma*.

[10] Risi E., Iacovelli R., Altavilla A. (2013). Clinical and Pathological Features of Primary Neuroectodermal Tumor/Ewing Sarcoma of the Kidney. *Urology*.

[11] Friedrichs N., Vorreuther R., Poremba C. (2002). Primitive Neuroectodermal Tumor (PNET) in the Differential Diagnosis of Malignant Kidney Tumors. *Pathology-Research and Practice*.

[12] Das D., Datta C., Pal D. (2020). Primary Ewing Sarcoma of the Kidney in an Adult and a Child: Solving a Diagnostic Challenge. *Indian Journal of Pathology & Microbiology*.

[13] Womer R. B., West D. C., Krailo M. D. (2012). Randomized Controlled Trial of Interval-Compressed Chemotherapy for the Treatment of Localized Ewing Sarcoma: A Report From the Children’s Oncology Group. *Journal of Clinical Oncology*.

[14] Abboud A., Masrouha K., Saliba M. (2021). Extraskeletal Ewing Sarcoma: Diagnosis, Management and Prognosis (Review). *Oncology Letters*.

